# Fatigue and Its Contributing Factors in Chinese Patients with Primary Pituitary Adenomas

**DOI:** 10.1155/2023/9876422

**Published:** 2023-03-15

**Authors:** Xiaomei Zhang, Yanqing Li, Dandan Zhang, Yueping Zhong, Tian Li

**Affiliations:** ^1^Department of Neurosurgery, Affiliated Hospital of Nantong University, 20th Xisi Road, Nantong 226001, China; ^2^Medical College, Nantong University, 19th Qixiu Road, Nantong 226001, China; ^3^Department of Nursing, Nantong Health College of Jiangsu Province, 288th Zhenxing East Road, Nantong 226010, China; ^4^Department of Nursing, Affiliated Hospital of Nantong University, 20th Xisi Road, Nantong 226001, China; ^5^School of Basic Medicine, Fourth Military Medical University, No. 169 Changle West Rd, Xi'an 710032, China

## Abstract

**Background:**

Pituitary adenomas (PAs) refers to a group of benign tumors that develop in the pituitary gland and are often characterized by fatigue. However, fatigue has not been documented in any Chinese research involving people with primary PA. The study sought to examine the prevalence, predictors, and correlation of fatigue with the quality of life (QoL) among PA patients in China.

**Methods:**

In total, 203 primary PA patients were included in this cross-sectional study. A series of questionnaires were administered, including the Multidimensional Fatigue Inventory (MFI), M. D. Anderson Symptom Inventory Brain Tumor (MDASI-BT), Short-Form 36 Health Survey (SF-36), Pittsburgh Sleep Quality Index (PSQI), and the Hospital Anxiety and Depression Scale (HADS). Data analysis was accomplished by Pearson or Spearman correlations, linear regression, and simple path analysis.

**Results:**

Severe fatigue prior to the initial diagnosis and preparation for surgery affected 50% of PA patients. Depression, sleep disturbance, and MDASI-BT symptom total scores were independently able to predict patient fatigue. Sleep disturbance mediates the influence of depression on fatigue (IE sleep = 0.296, 95% CI: LB = 0.148 to UB = 0.471).

**Conclusions:**

Chinese patients with primary PA often report experiencing fatigue. Depression and poor sleep quality were shown to be significant contributors to PA patients' fatigue. Depression affects PA patients' fatigue directly or indirectly. Medical professionals should take a proactive approach to PA patients suffering from fatigue before initial diagnosis and preoperative preparation to determine necessary interventions early, thus reducing fatigue and ultimately enhancing their QoL.

## 1. Introduction

Pituitary adenomas (PA) are relatively common, representing 10% to 15% of all intracranial tumors; their reported prevalence rates range between 10% and 22% [1, 2]. According to their endocrine functions, PA is classified into nonfunctional and functional types. The main clinical manifestations of nonfunctional PA are various symptoms caused by pituitary compression or optic chiasm [3] (e.g., headache [4] and diminution of vision [5]). Additionally, some patients will experience pituitary dysfunction, nerve paralysis, or a pituitary stroke [6]. Functional PA can lead to a range of hormonal abnormalities as follows [7]: prolactin, growth hormone, follicle-stimulating hormone adenomas, luteinizing hormone, thyrotropic hormone, and adrenocorticotropic hormone. Patients with functional PA experience several endocrine symptoms due to the abnormal secretion of the pituitary hormone. Different types of pituitary tumors have varying characteristics and clinical manifestations. These diverse clinical manifestations affect physical functions and cause great damage to the patient's psychological and social functions. Quality of life (QoL) reflects the QoL of patients [8–10]. Several studies show that PA patients have a low QoL, which differs significantly from that of healthy people [11, 12].

When interpreting how fatigue is experienced, a dualistic answer is typically received. Some diseased individuals describe it as “restful tiredness,” “active exhaustion,” or just a general “vitality loss.” Fatigue influences the complete lifestyle of an individual with the adoption of sedentary characteristics, including metabolic changes, depression, poor dietary intake, and anxiety [13, 14]. Fatigue, which has been known to alter cancer prognosis, represents a complex interaction of behavioral, social, disease, biological, and demographic processes [15, 16]. The evidence gathered from clinical practice reveals that fatigue plays an integral function in the development and recovery of brain tumor patients. It has been estimated that 89%–94% of patients with recurrent malignant gliomas have cancer-related fatigue (CRF) when assessed using verified and robust methods for this group [17]. Despite completing treatment, 39% of patients with low-grade gliomas still experienced fatigue up to 8 years later [18]. Fatigue is ranked as more disconcerting than other symptoms, including pain, nausea, and vomiting, all of which may be treated with medicine [19]. PAs, unlike malignant gliomas, have not been the subject of the extensive research that has explored the causes of fatigue in individuals with primary brain tumors. In general, the degree of preoperative fatigue in patients with pituitary tumors and its influencing factors remain unknown [20–22].

A survey on sleep quality in PA patients showed that severe anxiety and depression 3 months after surgery were significantly related to their sleep quality [23, 24]. Dutch researchers found that patients with PA had symptoms of physical pain, sleep problems, cognitive problems, decreased libido, depression, and increased sensitivity to stress [25]. Complete surgical excision is not usually accomplished despite the benign and slow-growing nature of PA tumors; in addition, 6–30% of adenomas continue to progress following subtotal resection [26]. Patients with pituitary tumors may experience adverse conditions that lead to a decline in their QoL before and after treatment [27]. Due to the co-occurrence of these symptoms, patients with PA may also experience chronic fatigue.

However, fewer studies have focused on fatigue in PA patients to the same extent as other tumor types, especially gliomas. For these studies, firstly, for fatigue in PA patients, fatigue symptoms were only mentioned in the assessment of QoL. In most cases, just few questions in QoL instrument are used for evaluating fatigue in PA patients [11]. Seventeen patients with nonfunctioning pituitary macroadenomas who were treated for their condition experienced poor sleep quality, considerably impaired QoL, and subjective fatigue [28]. Fatigue is a multidimensional notion with a variety of manifestations (affective, cognitive, and physical aspects); hence, a complete assessment strategy ought to include a multidimensional instrument [29]. Second, the neurological features, therapy, pathophysiology, causes, and processes of PA are distinct from those of other intracranial malignancies. Moreover, neuro-oncological research has diagnosed and measured fatigue in a rather simplistic manner, employing the visual analogue scale (VAS) or a single fatigue item in the instrument. Given that fatigue is a multidimensional problem, a comprehensive evaluation of patients with pituitary tumors may yield new results. Thirdly, fatigue in PA patients may be influenced by abnormal hormone levels, which differ from other types of brain tumors [30, 31]. Furthermore, especially in cross-sectional research, baseline data are commonly lacking. Additionally, although fatigue is often considered a treatment-related side effect, its pretreatment state or association with tumor-related symptoms is unknown. Generally, there has been no documentation of pretreatment evaluations of fatigue in PA patients.

This study aimed to (1) examine primary PA fatigue status in Chinese patients; (2) evaluate fatigue predictors in primary PA patients, and (3) explore the association of fatigue with the QoL of primary PA in this population. Additionally, the literature analysis reveals that researchers in the field of oncology have explored the link between poor sleep, depression, and fatigue [32–34]. Therefore, we tested the hypothesis that the quality of sleep has a mediating effect on the link between depression and fatigue.

## 2. Methods

### 2.1. Study Design and Setting

The Ethics Committee of the Affiliated Hospital of Nantong University granted its approval for this single-center cross-sectional research (2019-K003). Informed consent forms were obtained from all participants upon discussion of the study scope. Patients' baseline sociodemographic and clinical characteristic data were acquired. Self-reporting was utilized for patient sociodemographic information collection. Electronic medical records were accessed to obtain clinical data relating to hospitalization, hormone levels, and tumor size.

### 2.2. Participants

Between August 2018 and November 2020, patients admitted to our facility were recruited. Only 203 individuals with primary PA were included in the present research after excluding 5 patients who did not meet the eligibility requirements.

Participants were required to have met the following requirements to be included in the study: (I) patients older than 18 who underwent an endoscopic transsphenoidal PA excision; (II) conscious and sound of mind and body as well as having the ability to fill out the survey questionnaire; (III) articulate through verbal and written language with study personnel; and (IV) participants who agreed with the participation scope and provided informed consent.

The following conditions disqualify patients from participation in the research: (I) previous history of cancerous tumors other than PA; (II) a history of psychosis or other disorders that might influence the assessments of the therapeutic outcomes of PA resection; and/or (III) a history of acute heart failure, severe cerebrovascular sequelae, and/or acute infection, or having undergone a major surgical procedure.

### 2.3. Assessment Tools

PA Patients participating in the study completed a set of standardized self-report questionnaires (as detailed below).

#### 2.3.1. Clinical and Sociodemographic Characteristics

The demographic variables included sex, age, exercise therapy, place of residence, monthly per capita income, body mass index (BMI), marital status, education level, alcohol use, employment, tobacco use, and medical insurance.

Clinical data, including tumor size, comorbidity, and disease duration were collected based on the self-reporting data or through electronic medical records. Other clinical variables, such as thyroid-stimulating hormone, prolactin (PRL), total thyroid hormone, total triiodothyronine, free thyroid hormone, free triiodothyronine, cortisol, and adrenocorticotropic hormone levels were assessed during the study.

#### 2.3.2. Multidimensional Fatigue Inventory-20 (MFI-20)

The MFI-20, which contains 20 items, assesses fatigue in five different ways: general fatigue, mental fatigue, reduced motivation, reduced activity, and physical fatigue. Each dimension is measured on a scale of 1–20, with an increasing number positively correlated with fatigue and impairment levels. Scores on various dimensions were calculated as the sum of individual item totals; these scores were ranked and indicated various levels of fatigue from mild (5–8), moderate (9–12), severe (13–16) and very severe (17–20). The total score, which varied from 20 to 100, was derived by adding the scores from each of the five measures of fatigue. A score >60 was indicative of severe fatigue [35].

#### 2.3.3. M. D. Anderson Symptom Inventory—Brain Tumor (MDASI-BT)

The MDASI-BT measures the severity of symptoms and how much they interfere with everyday life. It has twenty-two symptoms in all (13 core and 9 specific symptoms for patients with brain tumors). Each symptom reported by the patient in the preceding 24 hours was rated on an 11-point scale (wherein 0 represents “not present” and 10 represents “as bad as you can imagine”) to indicate its presence and severity. Additionally, the MDASI-BT also rated how much these symptoms had hindered everyday activities in the preceding 24 hours. It comprises 6 interference items (3 activities and 3 mood interference items). The interference items were also measured on an 11-point (0–10) scale, wherein 0 represents “did not interfere” and 10 represents “interfered completely” [36].

#### 2.3.4. The Karnofsky Performance Status (KPS)

KPS is a subjective assessment of the overall performance status of a patient. It is assigned based on a scale of 0–100 in increments of 10 (with 0 representing death and 100 indicative of a patient in optimal health) [37].

#### 2.3.5. The Hospital Anxiety and Depression Scale (HADS)

HADS is a self-report tool designed to evaluate patients' states of depression and anxiety. Anxiety and depression are assessed using a 7-item subscale. Subscale scores are established through a summation for the range of 0–21. Anxiety and depression are considered clinically significant when a patient's score on at least one of the measures is 8 or larger [38].

#### 2.3.6. The Pittsburgh Sleep Quality Index (PSQI)

The PSQI was utilized to analyze the quality of sleep. The following are the seven aspects that make up the PSQI scale: daytime dysfunction, use of sleep drugs, sleep duration, sleep disturbances, subjective sleep quality, sleep latency, and sleep efficiency. Scores on the PSQI vary from 0 to 21, and bracketing occurs in intervals of 5 (0 inclusive). The sleep quality scores are ranked as follows: <4 (good), 5–10 (moderately good), 11–15 (moderately bad), and >16 (poor). In this research, poor quality of sleep was defined as a PSQI cumulative score of >5 [39].

#### 2.3.7. Short-Form 36 Health Survey (SF-36)

The SF-36 contains two high-order summary scales (physical and mental components, PCS and MCS, correspondingly) that were used to measure the general health status of each patient. Multifunction item scales and the summary measure scores ranged from 0 to 100, wherein a high score was positively correlated with a better QoL [40].

### 2.4. Data Collection Procedure

To guarantee that the survey findings are objective and reduce the impact of surveyors' subjective bias, we established standardized methods for inquiry and clarification of questionnaire items. An investigator asked the questions orally and administered the questionnaire to patients who did not know their diagnoses. For accurate data input, we double-checked the data at regular intervals.

### 2.5. Data Analysis

AMOS 26.0 and SPSS 24.0 were employed to process the data. The Kolmogorov–Smirnov test was utilized to examine the distribution of the data. The mean ± SD was adopted to present a normally distributed data set. Skewed distributions were described utilizing descriptive statistics, which were represented as medians (25th and 75th percentiles) or percentages. Statistical analysis was performed using the Pearson correlation if the two continuous variables of interest had a normal distribution and the Spearman rank correlation coefficient otherwise. The relationship between fatigue and QoL was investigated using the Spearman correlation analysis. Univariate factors with a *P* value < *0.05* were subjected to a multiple stepwise linear regression analysis to determine their predictive value for fatigue in PA patients. *P* < 0.05 (two-sided) was the significance threshold. Furthermore, we conducted a simple path analysis to determine the extent to which sleep mediates between depression and fatigue. In total, 2,000 bootstrap resamples were done to evaluate whether sleep quality was a mediator of the relationship between depression and fatigue. The confidence interval of the mediating effect was calculated by the bootstrapping method with bias correction. The figures and graphs were plotted using GraphPad Prism and RStudio.

## 3. Results

### 3.1. Patient Features

Five patients out of a total of 208 were not included since they did not fill out their questionnaires. In the end, 203 participants were included in the analysis. Patients diagnosed with PA had a mean (SD) age of 54.51 ± 12.94 years, with 46% being male. Patients with PA had a median duration of 1.73 years following diagnosis, and 34.9% had concurrent conditions. During the course of the disease, the most common complaints were poor vision (56.16%), headache (30.5%), and dizziness (33.8%). These PA patients could be classified as having either a macroadenoma phenotype (93.8%), a giant phenotype (1%), or a microadenoma phenotype (5.2%). Fifteen percent of patients exhibited aberrant prolactin levels. The median KPS score of the PA patients was 90. Tables 1 and 2 provide detailed summaries of the psychological, clinical, and demographic features of PA patients.

### 3.2. Fatigue Prevalence and Severity Among Primary PA Patients

**Figure 1:**
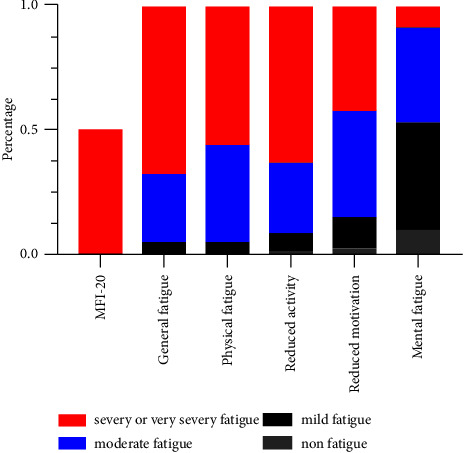
The proportion of fatigue with different severity in patients (*N* = 203), measured with the MFI-20 questionnaire.

The proportion of fatigue, along with its varying severity in patients is shown in Figure 1. The median (IQR) multidimensional fatigue scores on the MFI-20 instrument were specified as follows: general fatigue, 14 (12, 15); physical fatigue, 13 (11, 15); reduced activity, 13 (12, 15); reduced motivation, 12 (10, 14); mental fatigue, 8 (6, 11); and total fatigue, 61 (54, 67), respectively. As per all the subscales of the MFI-20 questionnaire, up to 67.5% of participants developed severe fatigue in the general fatigue subscale, followed by the reduced activity subscale (62.6%), physical fatigue scale (56.2%), reduced motivation subscale (42.4%), and mental fatigue scale (8.9%). Moreover, a total fatigue score > 60 was observed in 102 individuals, and severe total fatigue had a prevalence of 50.2%.

### 3.3. Association of Clinical, Demographic, and Psychological Factors with Fatigue in Individuals with Primary PA

Patients in primary PA had their clinical, psychological, and demographic features correlated with fatigue as per Spearman rank correlation coefficients derived. Old age (*P*=0.001; *r* = 0.231) and employment (*P*=0.005; *r* = −0.196) were substantially associated with the total fatigue score. Old age was also correlated with higher scores across the three dimensions of the MFI-20 scale (i.e., general fatigue *P*=0.034; *r* = 0.149, reduced activity (*P*=0.044; *r* = 0.142), and mental fatigue (*P* < 0.001; *r* = 0.310). Employment was also negatively correlated with two dimensions of the MFI-20 scale (i.e., general fatigue *P*=0.043; *r* = −0.142 and mental fatigue *P*=0.002; *r* = −0.214). Additionally, less educated patients showed higher mental fatigue scores (*P*=0.001; *r* = 0.222). In addition, exercise adherence was inversely linked to the total fatigue score (*P*=0.018; *r* = −0.166). Patients who did not exercise had higher general fatigue (*P*=0.038; *r* = −0.146), reduced motivation (*P*=0.011; *r* = −0.178), and mental fatigue scores as compared to those who exercised (*P*=0.017; *r* = −0.167), as shown in Table 1.

Table 2 depicts positive links between anxiety/depression, and total fatigue score (*P* < 0.001; *r* = 0.634, and *r* = 0.626, correspondingly) and the five dimensions. A lower KPS score was substantially linked to the total fatigue score (*P* < 0.001; *r* = −0.320) and the five dimensions (*P* < 0.001, *r* = −0.264; *P*=0.001, *r* = −0.230; *P*=0.002, *r* = −0.217; *P* < 0.001, *r* = −0.311; and *P*=0.023, *r* = −0.160, respectively). Poor sleep quality was related to the total fatigue score (*P* < 0.001; *r* = 0.486) and each dimension individually (*P* < 0.001; *r* = 0.548, *r*=0.392, *r*=0.487, *r*=0.242, and *r* = 0.302, correspondingly). Additionally, patients with higher MDASI.BT total symptom scores (*P* < 0.001; *r* = 0.657, *r*=0.539, *r*=0.539, *r*=0.637, *r*=0.474, and *r*=0.363, respectively) and counts had higher total fatigue scores (*P* < 0.001; *r* = 0.557, *r* = 0.450, *r* = 0.455, *r* = 0.552, *r* = 0.313, and *r* = 0.420, respectively). Abnormal PRL was significantly correlated with higher scores across the three dimensions of MFI-20 (i.e., general fatigue, physical fatigue, and reduced activity) (*P*=0.010; *r* = 0.182; *P*=0.009, *r* = 0.185, and *P*=0.048, *r* = 0.141, respectively).

### 3.4. Depression, Sleep Disturbance, and Higher MDASI.BT Total Symptom Scores Predict Fatigue in Primary PA Patients

A multiple stepwise linear regression analysis was employed to thoroughly examine the detected probable fatigue factors. As depicted in Table 3, the results indicated that depression (*β* = 0.243; *P*=0.012), sleep disturbance (*β* = 0.201; *P* < 0.001), and MDASI.BT (*β* = 0.284; *P*=0.001) scores were potentially significant predictors of fatigue in primary PA patients.

### 3.5. Pathway Analysis for Sleep, Depression, and Fatigue

**Figure 2:**
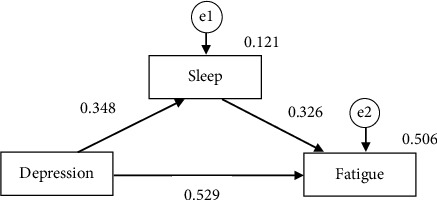
Standard coefficients of the mediator model.

A strong direct relationship was discovered between the three factors in the pathway analysis. The link between depression and fatigue was mediated by sleep disturbance. The direct effect of depression on sleep disturbance was 0.348 (*P* < 0.001). On the other hand, the direct effect of sleep disturbance on fatigue was 0.326 (*P* < 0.001). Also, the direct effect of depression on fatigue was 0.529 (*P* < 0.001). There was a statistically remarkable indirect influence of depression on fatigue through the pathway of sleep disturbance (IE sleep = 0.296; 95% CI: LB = 0.148 to UB = 0.471) (Table 4, Figure 2).

### 3.6. Correlation Between Fatigue and QoL in Primary PA Patients

**Figure 3:**
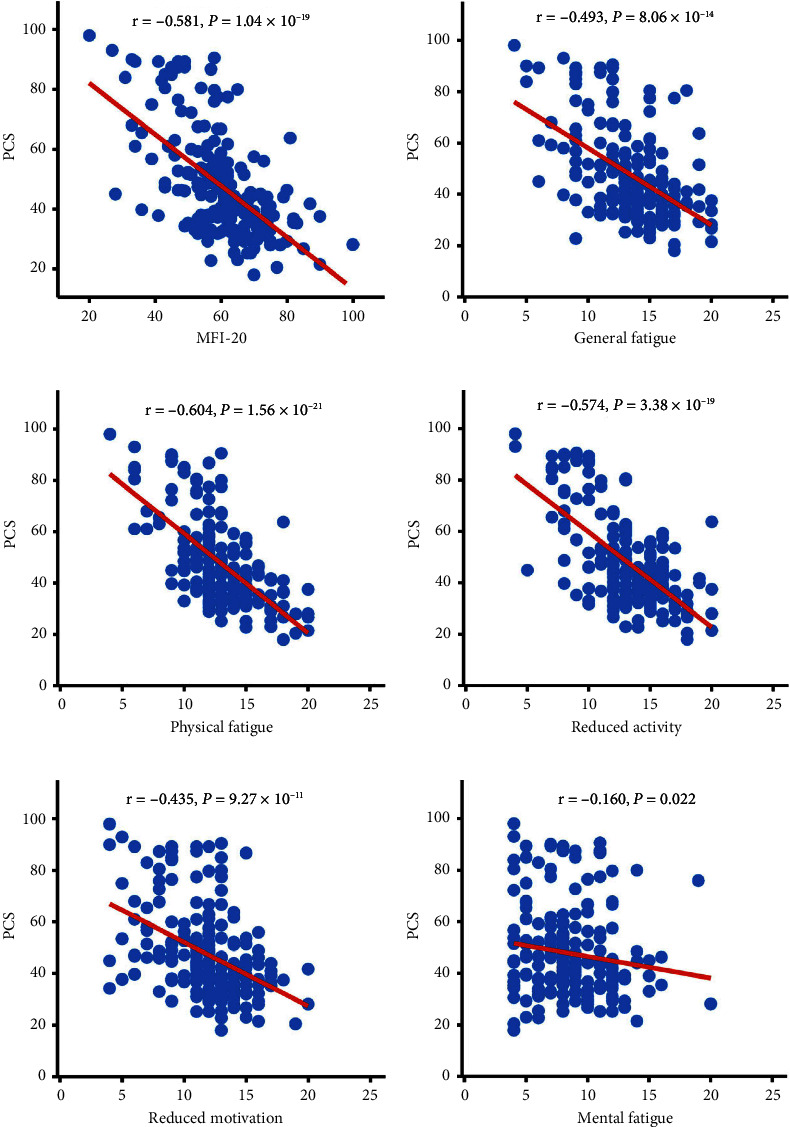
Correlation between fatigue and quality of life (PCS) in patients with pituitary adenoma. (a) Correlation between PCS and MFI-20. (*r* = −0.581; *P* < 0.01). (b) Correlation between PCS and general fatigue. (*r* = −0.493, *P* < 0.01). (c) Correlation between PCS and physical fatigue. (*r* = −0.604; *P* < 0.01). (d) Correlation between PCS and reduced activity. (*r* = −0.574; *P*  < 0.01). (e) Correlation between PCS and reduced motivation. (*r* = −0.435; *P* < 0.01). (f) Correlation between PCS and mental fatigue. (*r* = −0.160; *P* < 0.05). Abbreviations: PCS: physical component summary.

**Figure 4:**
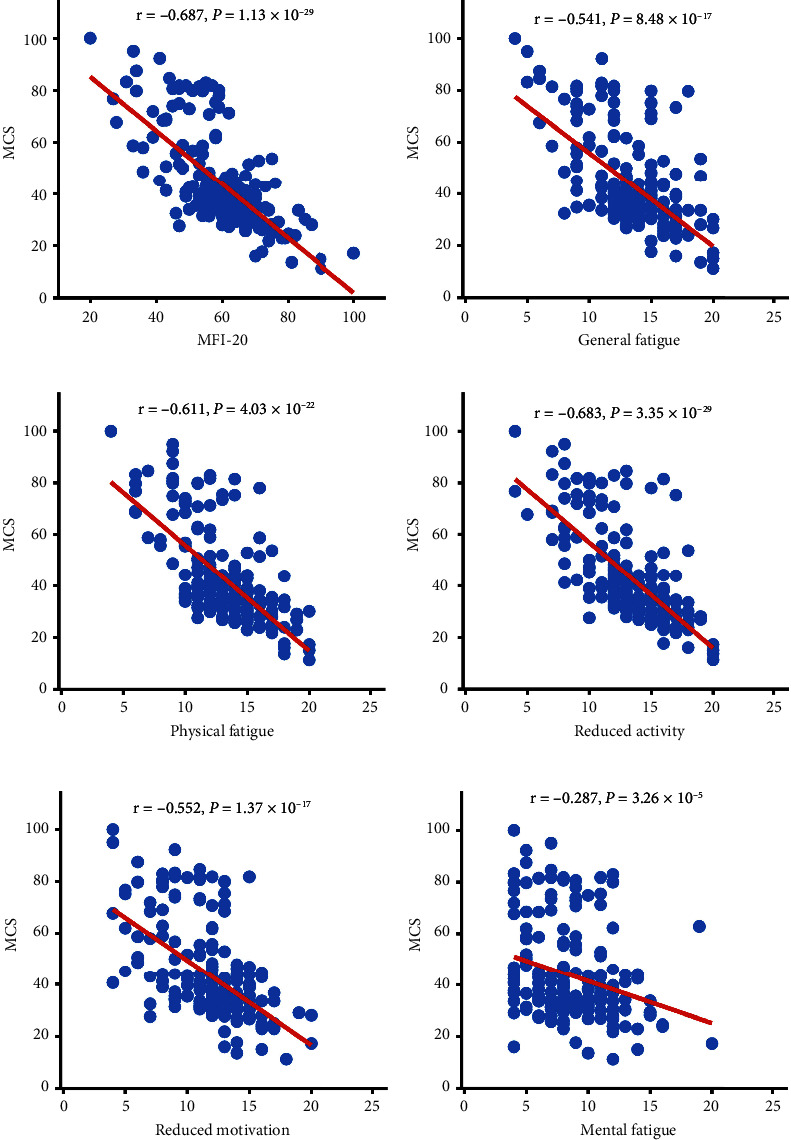
Correlation between fatigue and quality of life (MCS) in patients with pituitary adenoma. (a) Correlation between MCS and MFI-20. (*r* = −0.687; *P* < 0.01). (b) Correlation between MCS and general fatigue. (*r* = −0.541; *P* < 0.01). (c) Correlation between MCS and physical fatigue. (*r* = −0.611; *P* < 0.01). (d) Correlation between MCS and reduced activity. (*r* = −0.683; *P* < 0.01). (e) Correlation between MCS and reduced motivation. (*r* = −0.552; *P* < 0.01). (f) Correlation between MCS and mental fatigue. (*r* = −0.287; *P* < 0.05). Abbreviations: MCS: mental component summary.

Table 5 depicts the associations between the SF-36 and MFI-20 scores. There was a substantial link between the SF-36 Health Survey and each measure of QoL and total fatigue, physical fatigue, reduced activity, and reduced motivation. All dimensions of the SF-36 Health Survey were substantially linked to mental fatigue except for SF, BP, and PF. The occurrence of fatigue significantly reduced the QoL, both physically and psychologically, of the PA patients. Scatter plots of the correlation between fatigue and QoL (PCS and MCS) are shown in Figures 3 and 4.

## 4. Discussion

The present study examined fatigue and its correlated factors in primary PA patients and evaluated the correlation of fatigue with the QoL of PA patients. The results showed that fatigue in PA patients was significantly linked to sociodemographic characteristics of age, employment, and exercise, along with a range of self-reported variables (i.e., anxiety, depression, KPS score, sleep quality, severity, and several somatic symptoms). Depression, sleep disturbance, and MDASI. BT scores were significantly correlated with fatigue in PA patients. The link between depression and fatigue was mediated by sleep disturbance. Furthermore, fatigue significantly reduced the QoL, both physically and psychologically, of PA patients.

Our research found a correlation between fatigue and sociodemographic characteristics, even though these characteristics cannot be used as predictors. Notably, we found that age was remarkably linked to fatigue in PA patients. These findings are consistent with those of a cross-sectional study of primary brain tumors in America, which indicated that the prevalence of fatigue increased with age. Additionally, the association between fatigue and employment has been reported previously; Raymond reported that fatigue interfered with patients' ability to fulfill work roles and limited patients' daily living activities. In our study, employed PA patients likely suffer from more severe fatigue than unemployed PA patients. We also observed reduced fatigue and improved QoL in patients who exercised regularly. Regular exercise can substantially strengthen the muscles and enhance QoL [41]. Thus, clinicians should pay attention to older and employed populations. Furthermore, patients' lifestyle behaviors, such as their exercise routines, need to be evaluated more thoroughly. Teaching patients about exercise and ensuring that patients are aware of the importance and potential effectiveness of exercise in managing their disease may increase their motivation and awareness and optimize their health and well-being. In addition, abnormal PRL was correlated with fatigue. According to previous studies, abnormal PRL levels were associated with sleep and fatigue [42]. PRL is most likely involved in modulating the rapid eye movement sleep (REMS) cycle [43]. Thus, abnormal PRL can affect the patient's sleep cycle, thereby causing sleep morphology disturbances and resulting in fatigue. Although PRL was not a significant predictor, it is recommended that physicians provide those with aberrant PRL levels with special attention.

In this analysis, patients suffering from fatigue had higher scores of both HADS-A and HADS-D. We found a strong relationship between fatigue and depression among PA patients. Numerous emotional issues, such as anxiety, depression, behavioral disorders, and personality changes, may be linked to pituitary illnesses [44]. A previous study reported that 11.6% of PA patients had anxiety, and 30.9% had depression [45]. A study from Canada found that almost 40% of participants had clinically significant symptoms of depression. The conclusions of these studies confirm our findings. A prevalent problem for brain tumor patients, anxiety and depression, contributes to poor adherence to treatment, poor social functioning, impaired concentration, and insomnia. In addition, an ever-growing number of studies indicate fatigue risk is greatly enhanced by the psychological disorder's presence. It was further observed by multiple stepwise linear regression analyses that depression was a key part of fatigue. Similar associations have been identified in breast cancer [46], glioblastoma [22], and meningioma patients [47], but not in primary PA patients. We believe that these results show the increased need for patient systemic psychiatric screening and psychiatric management for PA patients. Additionally, we believe that this will help develop fatigue relief for PA patients by allowing for cognitive, behavioral, and emotional support strategies to allow for improved QoL. Mindfulness-based cognitive therapy is a tolerable and possibly beneficial treatment as evidenced by a previous study [48]. Patients with fatigued pituitary tumors could also gain from practicing mind-body therapies such as relaxation, music therapy, massage, touch therapy, and yoga.

Through multiple stepwise linear regression analyses, it was observed that sleep disturbance greatly affected fatigue levels. Sleep disturbance was positively correlated with fatigue scores and the 5 dimensions of MFI-20. Long-term sleep disturbances can lead to significant daytime dysfunction, poor mental health, and increased fatigue. Many studies have confirmed sleep disorder and fatigue interactions [49]. An analysis of the available research shows that PA patients experience impaired sleep quality [50]. A long-term follow-up of untreated acromegalic patients illustrated a decrease in the sleep quality of patients within one year after surgery, resulting in decreased QoL, greater drowsiness, and fatigue throughout the day [51]. Previously, the link between sleep disturbances and fatigue has been discovered in other conditions, including RA [52], among family members of children with cancer [53], and in patients with chronic spontaneous urticaria [54]. An important and common symptom of PA is increased sleep disturbance, which plays a key role in fatigue. In this study, most patients had difficulty falling asleep and had short sleep time, which suggests that we should give more psychological or drug support to improve patients' difficulty in falling asleep. Fatigue and sleepiness may be alleviated by taking restorative sleep measures. Thus, we should seek to actively evaluate and understand PA patients' sleep quality and develop various measures for fatigue relief via sleep quality improvement. Intervention alone may have no significant effect; therefore, multimodality treatment should be developed [55].

Additionally, somatic symptoms (including insomnia, distress, depression, and pain) have previously been associated with fatigue. These are believed to alter outcomes, such as functional status and perceived health. Several reports have shown a link between somatic symptoms and fatigue [56]. For example, previous scholars have found a direct positive link between fatigue and somatic symptoms. Somatization was shown to increase fatigue levels among relatively dysphoric individuals [57]. Our results suggest that somatic symptoms were substantially linked to fatigue in PA patients in terms of their overall performance status. The higher the total scores and symptom counts of patients with somatic symptoms, the higher the total fatigue scores as well as the scores across the 5 fatigue dimensions. Multiple stepwise linear regressions illustrated that the total symptom score was a strong predictor of fatigue. Thus, fatigue prevalence has great clinical implications as it is related to functional status impairment. We believe that clinicians should recognize the requirement for early and well-timed patient symptom assessments and related functional status for risk identification. Well-timed patient symptom assessments and targeted interventions require testing and the development of physical function improvement of patients. For example, interventions should be implemented to actively manage symptoms in the initial disease stages (e.g., by relieving headaches and vomiting). Scientific medication guidance should be given to patients, and personalized physical exercise plans should be developed in cooperation with rehabilitation physicians and others according to patients' symptoms and physical functions to help patients engage in disease self-management and improve their physical symptoms.

As we hypothesized, the direct effect of depression on fatigue was 0.529 (*P* < 0.001). This indicated that the severity of fatigue was proportional to the patient's degree of depression. Sleep has a positive mediating effect; the indirect effect of depression on fatigue through sleep was 0.296 (*P* < 0.001), which implied that poor sleep quality was correlated with elevated levels of depression, thereby resulting in increased fatigue. The present study's findings may shed light on the mechanisms through which depression exacerbates fatigue in PA patients. Therefore, physicians need to closely attend to those patients who are depressed and having trouble sleeping.

Finally, measuring health-related QoL is crucial in the overall patient health status assessment in PA patients. It has been observed in past studies that fatigue can be a recurrent or persistent symptom in patients with primary brain tumors that is not dependent on daily activities or alleviated adequately by rest [13, 15]. Notably, the negative effects of fatigue on a patient's QoL might be considerable. Fatigued patients, comparatively, have been found to suffer significantly greater anxiety, worse physical function, and an overall lower QoL [58]. This research also explored the link between fatigue and QoL, which is important since QoL is often used to assess patient outcomes. In this study, all SF-36 Health Survey domains were considerably linked to fatigue. The majority of the SF-36 Health Survey subscales have substantial correlations with the MFI-20-related five dimensions. Depression (self-reported), sleep disruption, and somatic symptoms were all linked to fatigue within our study group. Fatigue's impact on PA patients' mental health and disease conditions may exacerbate QoL declines. These findings support previous observations of patients with glioma [59] and findings in other patient populations [60, 61]. Though symptom expression may be related to shared neurobiological mechanisms such as inflammation and brain abnormalities, more extensive studies are required for these specific mechanisms in PA patients.

### 4.1. Strengths and Limitations

As the first research to quantify preoperative fatigue, our findings pave the way for proactive treatments to be taken preoperatively to reduce fatigue and enhance patient outcomes. However, some of the existing research only described the occurrence of fatigue as a component of patients' overall performance across various therapeutic trajectories. Patients with PAs often experience fatigue, but there has not been enough study to determine the prevalence, severity, or cause of this symptom. This is the first cross-sectional research in China to assess the impact of fatigue on the QoL of PA patients, to our knowledge. This study took into account a wide range of characteristics that have been linked to fatigue, laying the groundwork for future research and presenting evidence for the establishment of therapies for treating fatigue in PA patients.

Despite these strengths, our study has several limitations that warrant discussion. First, this study measured fatigue, QoL, psychological factors, and physical function using self-report questionnaires. Self-reported data are highly susceptible to bias, even though the tools employed in this research have been verified in brain tumor patients. Second, it was a single-center research study. Third, due to the cross-sectional nature of the research, we are unable to draw causal relationships between variables. Additional longitudinal studies of fatigue, QoL, and psychological status measures should be conducted at multi-centers to develop effective interventions to manage patients' fatigue and improve their QoL. Finally, further studies need to be conducted to identify potential pathophysiologic pathways that might affect fatigue in primary PA patients.

## 5. Conclusions

The part of this manuscript was published as a preprint in https://www.researchsquare.com/article/rs-1702253/v1 [62]. In summary, this research reveals a high prevalence of fatigue in PA patients who are newly diagnosed and preparing for surgical procedures, providing useful knowledge and insights into fatigue and QoL. Fatigue occurrence greatly reduced PA patients' physical and psychological QoL. Based on our findings, we believe that fatigue is multidimensional and has no single root cause. It plays a role in a greater network that is dependent on the severity of other symptoms and variables that may have various etiologies. Therefore, these factors including depression, sleep disturbance, and severe disease symptoms should be considered in future research when planning interventions and response assessments. The QoL of PA patients with fatigue may be improved if clinical practitioners devote more emphasis to these patients before the first diagnosis and preoperative preparation.

## Figures and Tables

**Table 1:** The relationship between fatigue and sociodemographic characteristics.

Variable	*N* (%)	MFI-20		General fatigue		Physical fatigue		Reduced activity		Reduced motivation		Mental fatigue	
		*r*	*P*	*r*	*P*	*r*	*P*	*r*	*P*	*r*	*P*	*r*	*P*
Gender, male^†^	94 (46.3)	−0.070	0.320	−0.063	0.371	−0.029	0.678	−0.049	0.492	−0.023	0.749	−0.099	0.159
Age^‡^	56 (46, 65)	0.231	**0.001**	0.149	**0.034**	0.125	0.075	0.142	**0.044**	0.129	0.068	0.310	<**0.001**
BMI^§^	24.71 ± 3.07	0.070	0.318	0.068	0.333	0.039	0.582	0.088	0.212	−0.013	0.853	0.010	0.884
Place of residence^†^		−0.001	0.984	−0.054	0.448	−0.098	0.165	0.048	0.495	0.009	0.899	0.077	0.278
Urban	144 (70.9)												
Rural	59 (29.1)												
Marital^†^		0.027	0.703	0.022	0.751	−0.004	0.951	0.034	0.626	0.013	0.851	0.008	0.914
Married	192 (94.6)												
Other	11 (5.4)												
Education level^†^		0.079	0.265	0.021	0.771	0.013	0.852	0.001	0.994	0.020	0.776	0.222	**0.001**
≤9 years	117 (57.6)												
>9 years	86 (42.4)												
Employment, yes^†^	120 (59.1)	−0.196	**0.005**	−0.142	**0.043**	−0.120	0.088	−0.134	0.056	−0.076	0.280	−0.214	**0.002**
Medical insurance, yes^†^	193 (95.1)	0.053	0.449	0.097	0.170	0.011	0.879	0.033	0.642	0.075	0.285	−0.007	0.923
Tobacco use, yes^†^	22 (10.8)	0.099	0.160	0.086	0.223	0.047	0.507	0.126	0.073	0.107	0.130	0.042	0.553
Alcohol use, yes^†^	21 (10.3)	0.033	0.636	0.095	0.179	0.020	0.780	−0.005	0.940	−0.018	0.799	0.080	0.255
Exercise adherence, yes^†^	18 (8.9)	−0.166	**0.018**	−0.146	**0.038**	−0.043	0.539	−0.079	0.262	−0.178	**0.011**	−0.167	**0.017**
Affordability of medical expenses^†^		0.106	0.132	−0.010	0.893	0.043	0.540	0.081	0.253	0.012	0.863	0.262	<**0.001**
Fully affordable	57 (28.1)												
Reluctantly affordable	120 (59.1)												
Serious straits	26 (12.8)												
Monthly per capita income^†^		−0.072	0.308	−0.020	0.782	−0.023	0.742	−0.054	0.446	0.009	0.901	−0.132	0.060
<¥3000	23 (11.3)												
¥3000–6000	126 (62.1)												
>¥6000	54 (26.6)												

Values highlighted in bold meet the criteria for statistical significance.^†^values are presented as the number (%) analyzed by Spearman correlation analysis.^‡^values are presented as the median (25th and 75th percentiles) and analyzed by Spearman correlation analysis.^§^values are presented as the mean ± SD and analyzed by Spearman correlation analysis. Abbreviations: BMI, body mass index; MFI-20, multidimensional fatigue inventory-20.

**Table 2:** The relations between clinical characteristics, self-reported variables, and fatigue.

Variable	*N* (%)	MFI-20		General fatigue		Physical fatigue		Reduced activity		Reduced motivation		Mental fatigue	
		*r*	*P*	*r*	*P*	*r*	*P*	*r*	*P*	*r*	*P*	*r*	*P*
Comorbidity, yes^†^	71 (35)	0.005	0.943	0.028	0.692	0.033	0.642	−0.033	0.636	−0.045	0.527	−0.009	0.899
Disease duration (year)^‡^	0.5 (0, 2)	−0.027	0.697	−0.011	0.881	−0.041	0.564	−0.040	0.573	0.046	0.519	−0.039	0.584
Tumor size^†^		−0.062	0.390	−0.017	0.819	0.039	0.592	−0.013	0.854	−0.101	0.163	−0.113	0.117
Microadenoma	10 (5.2)												
Macroadenoma	181 (93.8)												
Giant	2 (1)												
abn. PRL^†^	30 (15.1)	0.135	0.057	0.182	**0.010**	0.185	**0.009**	0.141	**0.048**	0.044	0.539	−0.042	0.554
abn. ACTH^†^	61 (36.3)	−0.110	0.156	−0.027	0.726	−0.104	0.180	−0.108	0.165	−0.048	0.536	−0.119	0.125
abn. cortisol^†^	52 (40.6)	−0.034	0.706	−0.034	0.706	0.012	0.897	0.014	0.874	−0.005	0.959	−0.076	0.396
abn. FT3^†^	20 (10.4)	0.022	0.765	0.038	0.604	0.008	0.915	0.049	0.501	−0.014	0.844	0.011	0.885
abn. FT4^†^	52 (27.1)	0.046	0.526	0.018	0.802	0.041	0.571	0.094	0.195	−0.002	0.975	0.055	0.446
abn.TT3^†^	39 (20.9)	0.080	0.275	0.049	0.505	0.036	0.624	0.107	0.146	0.050	0.497	0.074	0.315
abn.TT4^†^	34 (18.3)	−0.076	0.305	−0.042	0.569	−0.137	0.063	−0.040	0.589	−0.082	0.268	−0.017	0.814
abn. TSH^†^	24 (12.7)	0.052	0.476	0.081	0.269	0.013	0.857	0.067	0.362	0.080	0.274	−0.074	0.313
HADS-a^§^	9.46 ± 4.50	0.634	<**0.001**	0.589	<**0.001**	0.589	<**0.001**	0.555	<**0.001**	0.444	<**0.001**	0.348	<**0.001**
HADS-d^‡^	10 (7, 13)	0.626	<**0.001**	0.600	<**0.001**	0.608	<**0.001**	0.552	<**0.001**	0.462	<**0.001**	0.268	<**0.001**
KPS^‡^	90 (80, 90)	−0.320	<**0.001**	−0.264	<**0.001**	−0.230	**0.001**	−0.311	<**0.001**	−0.217	**0.002**	−0.160	**0.023**
PSQI^‡^	5 (3, 8)	0.486	<**0.001**	0.548	<**0.001**	0.392	<**0.001**	0.487	<**0.001**	0.242	<**0.001**	0.302	<**0.001**
MDASI.BT^§^													
Total symptom score	67.81 ± 32.64	0.657	<**0.001**	0.539	<**0.001**	0.539	<**0.001**	0.637	<**0.001**	0.474	<**0.001**	0.363	<**0.001**
Total symptom count	13.83 ± 4.73	0.557	<**0.001**	0.450	<**0.001**	0.455	<**0.001**	0.552	<**0.001**	0.313	<**0.001**	0.420	<**0.001**

Values presented as bold data are considered statistically significant.^†^values are presented as the number (%) analyzed by Spearman correlation analysis.^‡^values are presented as the median (25th and 75th percentiles) and analyzed by Spearman correlation analysis.^§^values are presented as the mean ± SD and analyzed by Spearman correlation analysis. Abbreviations: abn, abnormal; PRL, prolactin; ACTH, adrenocorticotropic hormone; FT3, free triiodothyronine; FT4, free thyroid hormone; TT3, total triiodothyronine; TT4, total thyroid hormone; TSH, thyroid-stimulating hormone; HADS-a, hospital anxiety and depression scale-anxiety; HADS-d, hospital anxiety and depression scale-depression; KPS, Karnofsky performance status; PSQI, Pittsburgh sleep quality index; MDASI.BT, M. D. Anderson symptom inventory brain tumor module; MFI-20, multidimensional fatigue inventory-20.

**Table 3:** Multiple stepwise linear regression analysis of fatigue in patients with pituitary adenoma.

	B (SE)	*β*	*t*	(95% CI)	*P* value	Model summary
HADS-d	0.641 (0.253)	0.243	2.534	(0.142, 1.140)	0.012	*F* *=* 31.237
PSQI	0.688 (0.174)	0.201	3.965	(0.346, 1.031)	<0.001	*P* < 0.001
MDASI.BT total symptom score	0.106 (0.032)	0.284	3.314	(0.043, 0.170)	0.001	Adjusted *R*^2^ = 60.7%

Abbreviations. SE: standard error; HADS-d, hospital anxiety and depression scale-depression; PSQI, Pittsburgh sleep quality index; MDASI.BT, M. D. Anderson symptom inventor brain tumor module.

**Table 4:** Path analysis on the direct and indirect effects of sleep quality on perceived depression and fatigue among patients with pituitary adenoma.

	Standardized *β*	SE	CR	*P* value
Direct path				
Sleep <--- depression	0.348	0.05	5.273	<0.001
Fatigue <--- depression	0.529	0.138	10.038	<0.001
Fatigue <--- sleep	0.326	0.18	6.182	<0.001

Indirect path	Standardized *β*	SE	CI	*P* value
Fatigue <--- sleep <--- depression	0.296	0.083	0.148 to 0.471	<0.001

SE, standard error; CR, critical ratio; CI, confidence interval.

**Table 5:** Correlation between fatigue and quality of life in patients with pituitary adenoma.

Dimensions	Scores	MFI-20	General fatigue	Physical fatigue	Reduced activity	Reduced motivation	Mental fatigue
PCS	43.75 (35.50, 55)	−0.581^*∗∗*^	−0.493^*∗∗*^	−0.604^*∗∗*^	−0.574^*∗∗*^	−0.435^*∗∗*^	−0.160^*∗*^
MCS	37 (32.25, 50.13)	−0.687^*∗∗*^	−0.541^*∗∗*^	−0.611^*∗∗*^	−0.683^*∗∗*^	−0.552^*∗∗*^	−0.287^*∗∗*^
PF	65 (50, 85)	−0.392^*∗∗*^	−0.306^*∗∗*^	−0.424^*∗∗*^	−0.437^*∗∗*^	−0.261^*∗∗*^	−0.058
RP	0 (0, 0)	−0.468^*∗∗*^	−0.329^*∗∗*^	−0.401^*∗∗*^	−0.470^*∗∗*^	−0.359^*∗∗*^	−0.198^*∗*^
BP	72 (41, 100)	−0.350^*∗∗*^	−0.346^*∗∗*^	−0.383^*∗∗*^	−0.360^*∗∗*^	−0.250^*∗∗*^	−0.075
GH	40 (35, 50)	−0.467^*∗∗*^	−0.442^*∗∗*^	−0.475^*∗∗*^	−0.405^*∗∗*^	−0.368^*∗∗*^	−0.159^*∗*^
VT	40 (35, 55)	−0.683^*∗∗*^	−0.614^*∗∗*^	−0.594^*∗∗*^	−0.689^*∗∗*^	−0.541^*∗∗*^	−0.288^*∗∗*^
SF	50 (50, 75)	−0.481^*∗∗*^	−0.372^*∗∗*^	−0.469^*∗∗*^	−0.485^*∗∗*^	−0.418^*∗∗*^	−0.120
RE	0 (0, 33.33)	−0.511^*∗∗*^	−0.362^*∗∗*^	−0.437^*∗∗*^	−0.477^*∗∗*^	−0.398^*∗∗*^	−0.232^*∗*^

^*∗∗*^Correlation is significant at the 0.01 level (2-tailed). ^*∗*^Correlation is significant at the 0.05 level (2-tailed). Abbreviations: PCS, physical component summary; MCS, mental component summary; PF, physical functioning; RP, role-physical; BP, bodily pain; GH, general health; VT, vitality; SF, social functioning; RE, role-emotional; MFI-20, multidimensional fatigue inventory-20.

## Data Availability

The data are available from the corresponding authors upon reasonable request.
